# Scaling the Children Immunization App (CIMA) to Support Child Refugees and Parents in the Time of the COVID-19 Pandemic: A Social Capital Approach to Scale a Smartphone Application in Zaatari Camp, Jordan

**DOI:** 10.1007/s44197-021-00029-x

**Published:** 2022-01-03

**Authors:** Yousef S. Khader, Wadih Maalouf, Mohammad Abu Khdair, Mohannad Al-Nsour, Eresso Aga, Adam Khalifa, Mohamad Kassasbeh, Soha El-Halabi, Tobias Alfven, Ziad El-Khatib

**Affiliations:** 1 Department of Community Medicine, Public Health and Family Medicine, Faculty of Medicine, Jordan University of Science and Technology, Irbid, Jordan; 2 Drug Prevention and Health Branch, Prevention Treatment and Rehabilitation Section, United Nations Office on Drugs and Crime, Vienna, Austria; 3 Global Health Development, The Eastern Mediterranean Public Health Network (GHD, EMPHNET), Amman, Jordan; 4 UNICEF, Amman, Jordan; 5 UNHCR, Amman, Jordan; 6 National Vaccination Program, Ministry of Health, Amman, Jordan; 7 Department of Global Public Health, Karolinska Institutet, Stockholm, Sweden; 8 Medical University of Vienna, Vienna, Austria

**Keywords:** Vaccination, COVID-19, Parenting skills, Refugees, Digital health, CIMA

## Abstract

**Background:**

Children vaccination is a key intervention for their survival, especially among refugees. Yet, children vaccination registration is done manually in refugees camps and there is no possibility to send reminders to parents to come back on time. We aimed to boost the parental registration of children’s vaccination records on a Children Immunization app (CIMA) while also availing the parents with useful parenting skills under COVID-19-related stress.

**Methods:**

We incorporated United Nations Office on Drugs and Crime (UNODC) Parenting Skills under COVID-19 information material, through CIMA in Arabic and English languages. We recruited 1100 children in February–March 2021, through a community health promotion dissemination approach. A team of two nurses from the local population and two volunteers (one trained nurse and one trained social worker), from the camp, was formed. They promoted the CIMA app at two clinics and through households visits in Zaatari refugee camp. Qualitative data on impressions and observations of the interactions with the Zaatari camp community were also collected.

**Results:**

A total of 1100 children, up to 15 months of age, eligible for vaccination were enrolled in CIMA, whereby the staff explained the content of the app in terms of vaccination schedule, health promotion materials for vaccination and parenting skills to their caregivers. During the household visits, the volunteers identified a total of 70 children that have incomplete history of vaccination records (*n* = 42/70 girls, 60%). Also, opportunities and challenges for scaling the app were documented.

**Conclusion:**

The scaling of CIMA as an innovative means of dissemination of risk and health information in challenging context such as refugee camps was feasible. In the context of vaccination needs for children, in refugee settings, such a need is more eminent, particularly in the context of COVID-19.

## Background

In March 2020, the first case of Coronavirus disease (COVID-19) was reported in Jordan. As of 17 March 2020, the government of Jordan has taken strict measures, including social and physical distancing, banning all forms of inbound, outbound movement or international travel, and ordering a national curfew to ensure complete country isolation [1]. Consequently, a lockdown was ordered on all border arrivals to Jordan from pandemic countries, and administrative governorates were isolated from each other. These pandemic preventive measures had a significant impact on refugees living in camps in Jordan, including triggering fear of losing community members, an increase in the level of anxiety, stress, depression, substance use, and violence against women and children. [2, 3].

Syrian refugees, in Zaatari refugee camp, are known to be vulnerable in terms of access to healthcare services and specially mental health support [4]. In March 2020, Jordan recorded its first case of COVID-19 in Mafraq governorate which includes the Zaatari refugees’ camp. This led to a curfew for the whole governorate, which has suspended mass preventive vaccination campaigns and prevented parents from visiting health facilities for vaccinations, especially in the early stages of the pandemic in Jordan. This problem was worrying as it did lead to a build-up of vulnerable individuals. The COVID-19 measure has contributed to the worsening of some mental illnesses or even the emergence of new ones in Syrian refugees and parents [5–7]. Some of these could be related to financial difficulties, as most workers in the camps stopped working. Others were related to uncontrollable behaviours of the children and increasing conflicts between parents and children [8, 9]. This could be explained by the fact that the COVID-19 pandemic led to increased interaction between adults and their children and parents had more time to monitor them. Additionally, the vaccination rate for refugees, prior to the COVID-19 pandemic, was approximately 25% [10] and the vaccination services got interrupted during the COVID-19-related lockdowns.

The Ministry of Health (MoH) of Jordan provides support for mental health services, for the refugees, provided by one psychologist, one psychiatrist and one were nurse that were available daily for mental health support using phone and onsite service [7]. Yet, there is a high need for a differential level of mental health support for refugees using smartphones. Due to the limited access to the camp, the need to create a safe space for dissemination and accessing reliable health information, a remote support service for the refugees can be a good solution to respond to their health needs. In general, the MoH of Jordan has been supportive for remote health solutions, such as telemedicine [11] and smart phone applications [12]. Additionally, the refugees are known to have a high level of technology literacy rate, including almost every family owns at least one Android smartphone [12]. With this in mind, we implemented an intervention study to support parents and caregivers in Zaatari camp during the COVID-19 pandemic using a leaflet on caregiving under COVID-19 developed by the United Nations Office on Drugs and Crime (UNODC) [13]. This leaflet was delivered using a smartphone application (app) called the Children Immunization App (CIMA) [5]. The CIMA is a vaccination reminder app that has already been implemented in Zaatari camp since 2019. This app includes (1) health education sheets provided by UNICEF, (2) registration of children’s vaccination schedule, (3) automated reminder for parents for the upcoming vaccination appointment for their children and (4) automated reminder for the parents in case they missed the vaccination appointment of their child [14, 15]. This report aimed at describing the dissemination of the UNODC parenting skills material through the CIMA app during the period of February through March 2021.

## Methods

### Refugees Need for Parenting Materials and CIMA

War exposure and forced displacement represents a direct threat to the wellbeing of caregivers and their children. A threat that is associated with many negative health and social consequences, this is further to financial loss, family separations or other emotional changes across family members. Nevertheless, the importance of engaged, responsive and stable parenting skills practices are valuable ingredients to protect children mental health, and across different cultural backgrounds (UNODC studies). Family remains the most important, and in many cases, the only, social institution around the lives of children in humanitarian and refugee settings. Despite the fact that caregivers’ nurturing skills are needed in such context more than ever, caregivers are often challenged with all the aforementioned factors making it a struggle for them to balance between their own emotional struggles and that of their children. Hence the need for specific tailored messages on means to improve communication, trust, problem-solving skills and conflict resolutions to help parents avail such much needed skills to their children. UNODC has documented the effectiveness of such strategies in such settings in multiple studies [16, 17]. The Zaatari camp represents one of the newest sites where an innovative means of introducing such experience was introduced.

The camp is considered one of the highly dense camps in the world and it hosts over 90,000 residents in 5 Km [2] area. The original CIMA project is a multi-stakeholder project, where it includes different organizations that provide healthcare services for refugees (details published elsewhere [14, 15]).

Due to the lockdown, there was a risk for uncertainties whether the physical access to the camp will be interrupted. Therefore, in this new phase of the study, there was a need to incorporate parenting skills to support the parents with health education material during the period of lockdowns. Therefore, we incorporated the UNODC Parenting Skills material [13] into the CIMA app (these material are available in over 40 languages and they have been adapted for COVID-19 as well). We used the published material in Arabic and English languages. Then, we promoted these materials by recruiting 1,100 additional children during the period of two months (February and March 2021). The eligibility criteria included accepting to participate in the pilot, have one child qualified for vaccination and they have access to one smartphone at their household.

### Data Collection and Personnel

The CIMA app was previously disseminated in the form of a non-randomized trial study design in March 2019, and it recruited 1000 children in Zaatari camp (500 children in each study arm) [15]. Then, in this study, we documented the process of scaling the CIMA in a refugee camp, during February and March 2021. We collected the data by sharing impressions and observations of the interactions with the community at the Zaatari camp. A team of two staff and two volunteers was formed to promote the CIMA app at two clinics and through household visits. The two volunteers were local refugees at the camp (one trained as a nurse and one trained as a social worker). They used facial masks to protect their nose and mouth, and they practiced physical distancing during their visits to ensure protection for both of parents and themselves. The team was trained by the staff from the ministry of health that was initially trained on CIMA app [14]. The team showed the parents how to install the app from the Google Play Store [18, 19]. Usually, parents have internet connection on their smartphones. When the internet connection was not stable, the team used internet machine (known by the name of internet bundle with a SIM card) to connect the smartphone to the internet to be able to download the app. Then the team explained the content of the app to the parents and showed them how to use it (this includes browsing the content and adding the vaccination history).

The CIMA was created using the service-dominant logic [20, 21]. This included collecting feedback from users and updating the app accordingly, using informal interviews [22, 23]. The stakeholders involved were the public health planners, local clinic staff, community volunteers, parents and children. Materials, from various international sources, that provide services to refugee children and caregivers were integrated and used (Table 1).

**Table 1:** Overview of the materials used and the sources

Material	Source
Vaccinations benefits	World Health Organization [24] ECDC [25]
Health education about COVID-19 and its vaccinations	UNICEF [26]
Parenting skills for refugees during COVID-19	UNODC [13, 27]
Jordan national vaccination schedule	Ministry of Health, Jordan [28]

## Results

During the months of February to March 2021, the enrollment of the updated CIMA project was conducted at two vaccination clinics in Zaatari camp during the operating days of the camp (Sunday through Thursday) and on Saturdays through household visits by the volunteers. A total of 1100 children were enrolled in CIMA, where the staff have explained the content of the app including vaccination schedule, health promotion for the vaccination and parenting skills. Also, the parents were trained on how to use and browse through the app. The children were qualified for vaccination, so their age varied between newborns and up to 15 months of age (no data were collected on the demographics of the rest of the children in the households).

On Saturdays, the volunteers were visiting the households in their neighbourhood to register children. During the household visits, the volunteers received more detailed questions from parents regarding the vaccinations. The three main categories of questions were (1) “Are children born with genetic disorders are eligible to receive vaccinations?”, (2) “What are the trustworthy internet sources when it comes to COVID-19 and COVID-19 vaccinations?” and (3) “Is it possible to add information about COVID-19 inside the CIMA app?”. These targeted discussions with caregivers led to identification of a total of 70/1,100 children that have an incomplete or no history of vaccination records. According to the parents, the children were born with genetic disorders and the parents assumed their children were not eligible for vaccination (*n* = 42/70 girls; 60%).

In addition to registration of children and inquiry about children’s vaccination status and, the CIMA app content was updated with information related to COVID-19 and vaccination (Table 1).

### Affinity and Appreciation of the App as a Health Promotion Tool

The staff observed a positive receptivity of the CIMA app as a health promotion tool. The parents were keen to be enrolled. Therefore, the objective of recruiting over 1000 children in the app was achieved in less than two months during the winter season. This was considered as a major milestone for the scalability of the app.

The parents expressed their trust in the app, to the staff, due to its clear privacy statement during installation. The engagement of locals from the camp and Ministry of Health staff have also increased trust in the app. Further, Syrian refugees’ parents have been reported to have a strong attachment to their children, including their health and wellbeing, due to the forced migration they have been exposed to it [6].

They requested to add more information about COVID-19 and its vaccination for the adults. Therefore, this was an encouraging finding showing that the parents and care givers were interested in learning more about COVID-19 vaccinations using the app. The parents appreciated parenting skills materials to support them, particularly during the lockdowns. These results show the added value for the app and indicate that digital solutions can be used to provide access to critical health information to hard-to-reach vulnerable populations such as the refugees’ parents and their children.

Also, previous studies documented increased risk of mental health problems among camp refugees and significant barriers for Syrian refugees seeking mental health treatment; this demonstrated that the Syrian camp refugee in Jordan continues to face significant stressors including symptoms of distress and reduced functioning in people’s daily activities due to their experience of emotional distress [9]. Shortage of mental health professionals, medication shortages, poor awareness of available mental health services, limited or absent screening protocols, and the financial hardship of refugee patients are significant limiting factors to accessing mental healthcare [29]. Therefore, the integration of the UNODC’s parenting skills material is timely and useful [13, 27].

### Field Challenges

The field experience that was shared by the staff was as follows: (i) the internet connectivity inside the camp was not reliable, especially during the heavy winter weather conditions; therefore, the staff did use a 3G card to facilitate the app download; (ii) access to the clinics was interrupted during heavy winter days, where the streets in the camp got flooded and muddy. Therefore, the staff decided to do household visits on Saturdays; and (iii) there is a growing proportion of non-Android users inside the camp using mainly Chinese-made smartphones with an operating system, which is not compatible with Android.

### Confidentiality and Data Security

The parents had a concern about whether the CIMA app can exchange information or interact with other applications on their smartphones. The staff explained to them that the CIMA app does not read any content on their smartphones. In addition, the process of data recording, use and storage has been presented to all concerned stakeholders governing the Zaatari camp. The ethics and approvals were received from Jordan University of Science and Technology (JUST) [14]. Additionally, the Google PlayStore Protect has reviewed and approved the app and its content [19].

## Discussion

By using the service-dominant logic, we created the values of the app through the active participation of both the clinic staff and the parents. This collaborative work between all stakeholders, as active participants in the process of the health promotion was a key ingredient for a holistic approach to family-focussed public health promotion. Such an approach leads to a mutual “win–win” situation between all stakeholders, where everyone feels their input is being considered and all can see its impact [30]. Finally, we have observed a relationship of trust between the parents, clinics and the local volunteers. The element of trust is highly relevant in the context of the vulnerable refugees’ populations [31]. Muecke describes that refugees have a high level of uncertainty who to trust, when fleeing violence or persecution [32]. Additionally, the Secretary General of the United Nations announced that globally, we are observing a dangerous epidemic of misinformation since the Second World War, due to the COVID-19 pandemic [33]. The CIMA app became a trustworthy platform for parents to receive verified information about immunization, health promotion and parenting skills.

Overall, the promotion of digital health remains a challenge despite its high need. One of the major barriers to the scalability of digital health solutions is fragmentation and trust [34]. Therefore, we have adopted an inclusive approach for all stakeholders. The users reported on the issue of trust and data safety—important topics when it comes to digital health [34], especially among refugees. However, in this project, we have established a strong sense of trust due to the commitment of the Ministry of Health to provide vaccination and other healthcare services to the refugees. For example, Jordan was the first country in the world to vaccinate its refugees population against COVID-19 [35]. We have conducted a holistic approach to health promotion by educating refugees against each of the vaccine preventable diseases and the consequences of the COVID-19 pandemic.

## Future Directions

Inside the Zaatari camp: the parents are sharing locally the app among each other, by recommending it to each other. Also, there is a growing need to pilot the app in other resource-limited settings outside the refugee camps (e.g. in rural areas, among non-smartphone users where we will test an SMS-based modality). The app, and its concept, is becoming a platform for a holistic support for families (immunization reminders, immunization schedule tool, health education regarding immunization and trustworthy source for COVID-19 and COVID-19 vaccinations).

## Conclusion

The role of the vaccination app is growing, where it is becoming more inclusive to the health education needs of the parents and their children. Such progress was possible due to the growing trust between all engaged stakeholders in the process.

## Data Availability

Not applicable.

## Abbreviations

App: Smartphone application
COVID-19: Coronavirus disease
CIMA: Children Immunization App
MoH: Ministry of Health
UNODC: United Nations Office on Drugs and Crime
UNICEF: United Nations International Children's Emergency Fund
